# Efficacy of Dose Escalation of Oral 5-Aminosalicylic Acid for Ulcerative Colitis With a Mayo Endoscopic Subscore of 1: An Open Label Randomized Controlled Trial

**DOI:** 10.1093/ibd/izae088

**Published:** 2024-04-24

**Authors:** Tomohiro Fukuda, Yasuhiro Aoki, Hiroki Kiyohara, Ayumi Yokoyama, Atsushi Nakazawa, Yusuke Yoshimatsu, Shinya Sugimoto, Kosaku Nanki, Yohei Mikami, Kayoko Fukuhara, Shinta Mizuno, Tomohisa Sujino, Makoto Mutaguchi, Kaoru Takabayashi, Yuichi Morohoshi, Yasuo Hosoda, Haruhiko Ogata, Yasushi Iwao, Makoto Naganuma, Takanori Kanai

**Affiliations:** Division of Gastroenterology and Hepatology, Department of Internal Medicine, Keio University School of Medicine, 35 Shinanomachi, Shinjuku-ku, Tokyo, 160-8582, Japan; Department of Gastroenterology, Yokohama Municipal Citizen’s Hospital, Kanagawa, Japan; Division of Gastroenterology and Hepatology, Department of Internal Medicine, Keio University School of Medicine, 35 Shinanomachi, Shinjuku-ku, Tokyo, 160-8582, Japan; Division of Gastroenterology and Hepatology, Department of Internal Medicine, Keio University School of Medicine, 35 Shinanomachi, Shinjuku-ku, Tokyo, 160-8582, Japan; Center for Advanced IBD Research and Treatment, Kitasato University Kitasato Institute Hospital, Tokyo, Japan; Department of Gastroenterology, Tokyo Saiseikai Central Hospital, Tokyo, Japan; Department of Gastroenterology, Tokyo Saiseikai Central Hospital, Tokyo, Japan; Division of Gastroenterology and Hepatology, Department of Internal Medicine, Keio University School of Medicine, 35 Shinanomachi, Shinjuku-ku, Tokyo, 160-8582, Japan; Division of Gastroenterology and Hepatology, Department of Internal Medicine, Keio University School of Medicine, 35 Shinanomachi, Shinjuku-ku, Tokyo, 160-8582, Japan; Division of Gastroenterology and Hepatology, Department of Internal Medicine, Keio University School of Medicine, 35 Shinanomachi, Shinjuku-ku, Tokyo, 160-8582, Japan; Division of Gastroenterology and Hepatology, Department of Internal Medicine, Keio University School of Medicine, 35 Shinanomachi, Shinjuku-ku, Tokyo, 160-8582, Japan; Center for Preventive Medicine, Keio University School of Medicine, Tokyo, Japan; Division of Gastroenterology and Hepatology, Department of Internal Medicine, Keio University School of Medicine, 35 Shinanomachi, Shinjuku-ku, Tokyo, 160-8582, Japan; Center for Diagnostic and Therapeutic Endoscopy, Keio University School of Medicine, Tokyo, Japan; Center for Diagnostic and Therapeutic Endoscopy, Keio University School of Medicine, Tokyo, Japan; Center for Diagnostic and Therapeutic Endoscopy, Keio University School of Medicine, Tokyo, Japan; Department of Gastroenterology, Yokohama Municipal Citizen’s Hospital, Kanagawa, Japan; Department of Gastroenterology, National Hospital Organization Saitama Hospital, Saitama, Japan; Center for Diagnostic and Therapeutic Endoscopy, Keio University School of Medicine, Tokyo, Japan; Center for Preventive Medicine, Keio University School of Medicine, Tokyo, Japan; Division of Gastroenterology and Hepatology, Department of Internal Medicine, Keio University School of Medicine, 35 Shinanomachi, Shinjuku-ku, Tokyo, 160-8582, Japan; Division of Gastroenterology and Hepatology, The Third Department of Internal Medicine, Kansai Medical University, Osaka, Japan; Division of Gastroenterology and Hepatology, Department of Internal Medicine, Keio University School of Medicine, 35 Shinanomachi, Shinjuku-ku, Tokyo, 160-8582, Japan

**Keywords:** ulcerative colitis, inflammatory bowel disease, large intestine, colonoscopy, inflammation

## Abstract

**Background:**

Endoscopic healing is generally defined as Mayo endoscopic subscore (MES) ≤1 in ulcerative colitis (UC). However, patients with an MES of 1 are at higher relapse risk than those with an MES of 0. This study evaluated the therapeutic efficacy of proactive dose escalation of oral 5-aminosalicylic acid (5-ASA) in UC patients with an MES of 1.

**Methods:**

An open-label, randomized controlled trial was conducted in 5 hospitals between 2018 and 2022. Ulcerative colitis patients in clinical remission under oral 5-ASA therapy and diagnosed as having an MES of 1 were enrolled. Patients receiving maintenance therapy other than 5-ASA and immunomodulator were excluded. Patients were randomly assigned in a 1:1 ratio to receive either a dose-escalated (intervention) or constant dose (control) of 5-ASA. Concomitant immunomodulator was used as the stratification factor in the randomization. The primary end point was relapse within 1 year. The subgroup analysis was stratified for the use of immunomodulators.

**Results:**

The full analysis set included 79 patients (39 intervention and 40 control). Immunomodulators were used in 20 (25.3%) patients. Relapse was less in the intervention group (15.4%) than the control group (37.5%; *P* = .026). In the subgroup with concomitant immunomodulators, relapse was also less in the intervention group (10.0%) than the control group (70.0%; *P* = .020). In patients without immunomodulators, the difference was not significant between 2 groups (intervention, 17.2%; control, 26.7%; *P* = .53).

**Conclusions:**

Dose escalation of 5-ASA reduced relapse within 1 year in UC patients in clinical remission with an MES of 1.

**Key Messages**

**What is already known?**

Ulcerative colitis (UC) patients with a Mayo endoscopic subscore (MES) of 1 have a higher risk of relapse than those with an MES of 0; however, the significance of proactive intensification of treatment is unclear.

**What is new here?**

Increasing doses of oral 5-aminosalicylic acid (5-ASA) reduced relapse in UC patients in clinical remission with an MES of 1, and this intervention might be more efficacious in those with immunomodulators.

**How can this study help patient care?**

Dose escalation of 5-ASA could be considered for patients with an MES of 1 and in clinical remission.

## Introduction

Treat-to-target is a widely recognized strategy that is used to optimize the treatment for ulcerative colitis (UC).^1,2^ Endoscopic healing reduces relapse risk, surgical risk, and colitis-associated cancer risk and improves patient quality of life^3–6^; therefore, endoscopic healing is the gold standard for treat-to-target in the Selected Targets for Treatment of Inflammatory Bowel Disease II program.^2^

The Mayo endoscopic subscore (MES) is the most widely used tool to assess endoscopic activity in clinical trials.^7^ Currently, endoscopic healing is defined as an MES of 0 and 1.^8,9^ Several studies have shown that an MES of 1 is associated with a higher risk of relapse than an MES of 0,^10^ indicating an MES of 0 is the optimal target. However, guidelines do not explicitly state that therapeutic intervention for individuals with an MES of 1 should be carried out. Previously, we have shown that therapeutic intervention in UC patients in clinical remission with an MES of 1 reduced the rates of clinical relapse.^11^ However, therapeutic interventions and patient backgrounds were not fully adjusted because it was a retrospective study.

The efficacy of 5-aminosalicylic acid (5-ASA) as a maintenance therapy for the remission of UC has been demonstrated in several studies^12^; however, the efficacy of 5-ASA has not been reported when patients are limited to those with an MES of 1. A recent study suggested that properly optimizing the dose of 5-ASA may reduce relapses in newly diagnosed patients with mild to moderately active UC,^13^ although the optimal timing was not fully assessed, and the endoscopic activity was not limited to an MES of 1. We hypothesized that therapeutic intervention involving a dose escalation of 5-ASA would reduce future relapse in UC patients with an MES of 1. Therefore, the aim of this study was to investigate whether therapeutic intervention with increasing doses of oral 5-ASA improved the prognosis of UC patients in clinical remission with an MES of 1.

## Materials and Methods

### Study Design

This study was a multicenter, open-label, randomized controlled trial in 5 hospitals in Japan from March 2018 to June 2022. The inclusion criteria of this study were as follows: (1) diagnosed with UC; (2) aged 16 years or older; (3) treated with 5-ASA; (4) in clinical remission defined as a partial Mayo score ≤2 together with a rectal bleeding subscore of 0; and (5) undergoing colonoscopy between March 2018 and June 2021 with a confirmed MES of 1. Patients who met all the inclusion criteria were eligible. Patients using any topical agent or treated with maintenance therapy other than 5-ASA or thiopurine were excluded. There were no restrictions regarding comorbidities. Additional exclusion criteria are described in the Supplementary Appendix.

The trial hypothesis was the superiority of the therapeutic intervention. The necessary sample size was estimated using a previous study in which the relapse proportion within 1 year was 30% with therapeutic intervention and 50% without the intervention. The current study was designed to have a power of 80% and an error of 5%. The dropout rate was estimated at 20%; therefore, a final sample size of 220 patients (110 per group) was chosen. However, slower than anticipated recruitment meant that the trial steering committee discontinued enrollment on June 30, 2021, before 220 patients had been recruited. No interim analysis was carried out in this study. The extension of the patient enrollment due date from December 31, 2018, to June 30, 2021, was carried out with an amendment to the study protocol.

### Therapeutic Intervention

The therapeutic intervention in this study was the dose escalation of oral 5-ASA. Any kind of topical agent use was prohibited until the end of the study or relapse. In cases in which the dose of 5-ASA had already reached its upper limit in the specific formulation used but not in another formulation, the dose escalation was carried out by changing the 5-ASA formulation. The formulations of 5-ASA used in this study were time-dependent mesalazine, pH-dependent mesalazine, multi-matrix system mesalazine, and salazosulfapyridine. The incremental dose increases in 5-ASA were determined at the discretion of the physicians. In the control group, the dose of 5-ASA was not changed from the start date until the end of the observational period. If concomitantly used, immunomodulators were administered also at the constant dose throughout the study.

### Randomization and Masking

Eligible patients were randomly assigned (using a computer-generated, stratified randomization procedure) to either the group with or without therapeutic intervention. Patients assigned to the group without intervention were defined as the control group. Patients were randomized within 30 days from the confirmation of an MES of 1. The stratified factor in the randomization procedure was the concomitant use of immunomodulators at the time of randomization. Neither patients nor healthcare providers were masked in the randomization.

### Endoscopic Severity Scoring

Endoscopists inspected the entire colon using conventional white-light imaging. All endoscopists were experts in inflammatory bowel disease and were well trained in scoring MES. Endoscopic scores were assessed at colonoscopy. An MES of 0 was defined as normal or inactive disease, and an MES of 1 was defined as mild disease such as erythema, decreased vascular pattern, or mild friability.^7^ The MES and the ulcerative colitis endoscopic index of severity (UCEIS) were used to assess endoscopic severity. When collecting data for this study, 2 or more trained monitors reviewed the pictures of endoscopic examination independently and confirmed again as MES of 1 before enrollment.

### Primary End Point

The primary end point was relapse within 1 year. Patients were followed up to 1 year or until the time of relapse if it was within 1 year. Relapse was defined as meeting both of the following 2 criteria: (1) a partial Mayo score ≥3 or a rectal bleeding subscore ≥1; and (2) addition of any induction treatments for UC including topical agents. The primary end point was predefined in the study protocol.

### Secondary End Points

The secondary end points were adverse events, additional treatment, and predictive factors for relapse. For the safety evaluation, all adverse event reports were assessed throughout the study and were tabulated using the Common Terminology Criteria for Adverse Events version 5.0. A severe adverse event was defined as that requiring admission. For the exploration of predictive factors for relapse, age, sex, disease duration, disease extension, dose of 5-ASA, fecal calprotectin, and ulcerative colitis endoscopic index of severity score at baseline, concomitant use of immunomodulators, prior use of systemic steroids, and medication adherence were considered as the candidates. Medication adherence was measured by self-reported questionnaire with visual analog scale at every visit and averaged in each patient. Adverse events and additional treatment for relapse were predefined in the protocol. Regarding predictive factors for relapse, the dose of 5-ASA, fecal calprotectin, UCEIS score at baseline, and medication adherence were also predefined in the protocol, and others were investigated in a post hoc analysis.

### Statistical Analysis

The primary analysis population was the full analysis set (FAS), which was based on the intention-to-treat principle and enabled the exclusion of patients with no efficacy data after randomization. Summary statistical tables were prepared using frequencies and proportions for categorical data and median and interquartile range (IQR) for continuous variables. Baseline patient characteristics were compared with the Fisher exact test for categorical variables and the Mann-Whitney *U* test for continuous variables.

For the primary analysis, we assessed the relapse proportion within 1 year both in the therapeutic intervention group and the control group and analyzed the results with the χ^2^ test. The 95% confidence intervals (95% CIs) for relapse proportion were estimated using the Clopper–Pearson method. Relapse-free cumulative survival was also estimated with the Kaplan–Meier method, and the difference between the 2 groups was analyzed with the log-rank test. The cumulative relapse rates in the 2 groups were calculated as 100 person-years.

We conducted 2 sensitivity analyses. First, the primary end point was also analyzed using the Cochran–Mantel–Haenszel method by using immunomodulators as strata and estimating the odds ratio for relapse. Second, the definition of relapse was modified with 2-item patient-reported outcomes^14^ to reduce the risk of information bias by physician’s decisions. Briefly, a rectal bleeding subscore ≥1 or a stool frequency subscore ≥2, regardless of additional induction therapy, was treated as a relapse. The relapse proportion within 1 year was estimated both in the therapeutic intervention group and the control group and analyzed with the χ^2^ test.

For the secondary analysis, we conducted a subgroup analysis stratified for the use of immunomodulators. The relapse proportion within 1 year was compared between the therapeutic intervention group and the control group in each of the stratified groups using the Fisher exact test. The daily dose of immunomodulator was calculated as mg per kg of whole-body weight. In case of 6-mercaptopurine use, the dose was divided by a coefficient of 2.08 and converted to the equivalent pharmaceutical dose of azathioprine as previously reported.^15,16^ For another secondary analysis, we explored the predictors for relapse with the Cox proportional hazard model. As the sample size was not sufficient to adjust for all the predictive factors simultaneously in the multivariable-adjusted analysis, the therapeutic intervention was adjusted separately for each predictor. Furthermore, we investigated whether a change in the 5-ASA formulation (eg, Pentasa, Asacol, Lialda, and Salazopyrin) or an increment in the dose of 5-ASA was predictive for relapse in the subgroup of patients with therapeutic intervention. In this analysis, the increment in the daily dose of 5-ASA (Δ5-ASA) was classified into 2 groups: Δ5-ASA ≤1000 mg/day and Δ5-ASA >1000 mg/day. The relapse proportions within 1 year were compared with Fisher exact test, and univariable logistic regression analyses were conducted to investigate the associations between the change in 5-ASA formulation or Δ5-ASA, and relapse within 1 year.

In the secondary analysis, the subgroup analysis stratified by the use of immunomodulators was not originally included in the protocol. In addition, age, sex, disease duration, disease extension, concomitant use of immunomodulators, and prior use of systemic steroids were not originally included in the protocol when exploring predictors of relapse with the Cox proportional hazards model.

We also conducted a power analysis to calculate the actual power in the analyses for both the entire population (primary analysis) and subgroups with or without immunomodulators.

All *P*s were based on the 2-tailed hypothesis, and values less than 0.05 were considered statistically significant. Statistical analyses were performed using JMP version 16.0.0 software (SAS Institute, Cary, NC, USA) or Stata version 17 (StataCorp, College Station, TX, USA). GraphPad Prism version 9 (GraphPad Software Inc., La Jolla, CA, USA) was used for drawing graphs.

## Results

### Baseline Characteristics

**Figure 1.**
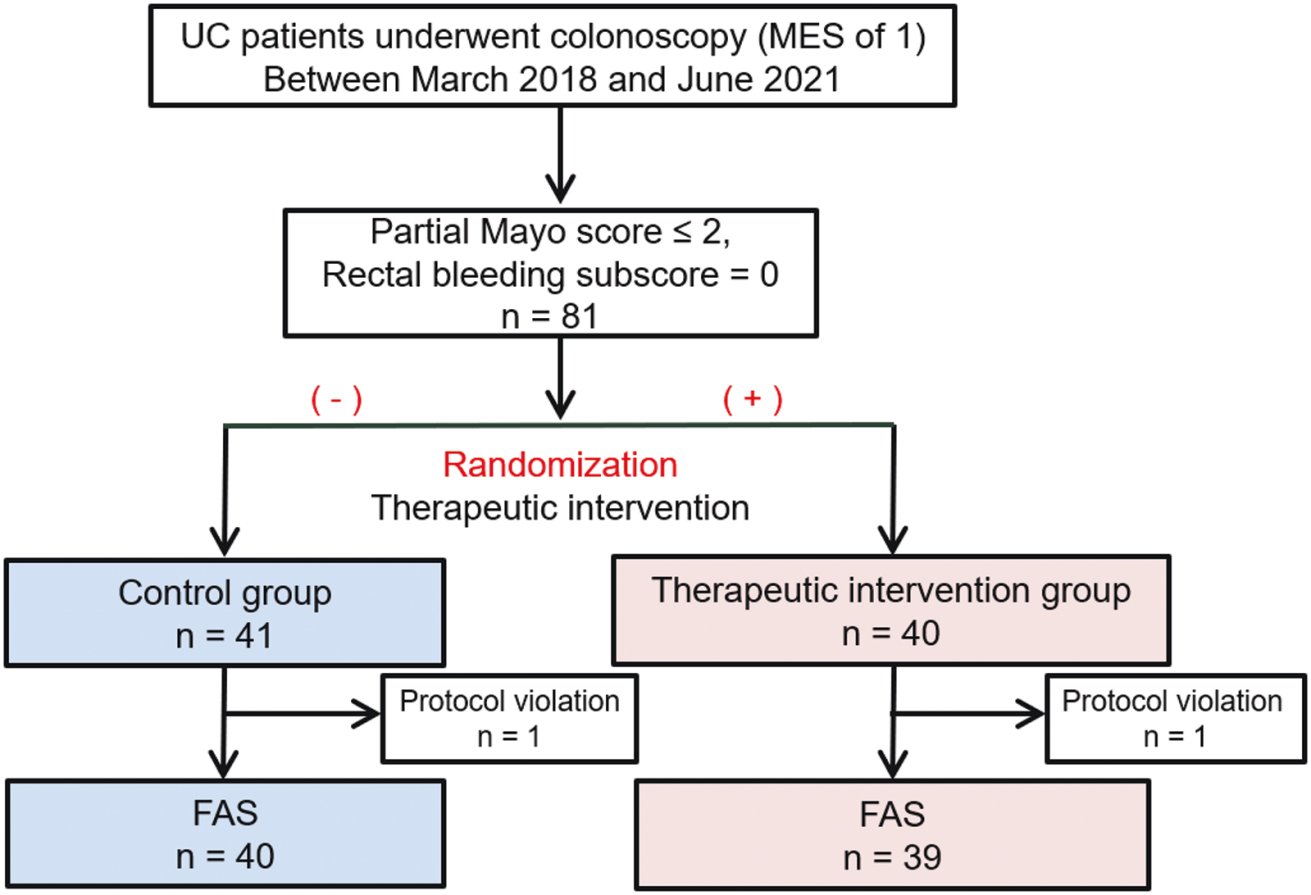
Flow chart of the study methodology. Ulcerative colitis (UC) patients with a Mayo endoscopic score (MES) of 1 who had a partial Mayo score ≤2 and a rectal bleeding subscore of 0 at first colonoscopy were included. A total of 81 patients were randomized (therapeutic intervention group, *n* = 40; control group, *n* = 41), and 79 patients were included in the full analysis set (FAS) after the exclusion of one patient from each group.

A total of 81 patients were randomized. However, 2 patients were excluded after randomization because of noncompliance with the first hospital visit or another protocol violation related to eligibility. Thus, 79 patients were included in the FAS for efficacy evaluation (Figure 1), including 39 in the therapeutic intervention group and 40 in the control group. A summary of demographic factors and baseline characteristics of the FAS population was shown in Table 1 and Supplementary Table 1. Detailed information on 5-ASA at baseline is presented in Supplementary Table 2.

**Table 1.** Patient characteristics at baseline.

Characteristic	Total *n* = 79	Control *n* = 40	Therapeutic intervention *n* = 39	*P*
Male ^a^	46 (58.2)	20 (50.0)	26 (66.7)	0.173 ^d^
Age, years ^b^	49 (39–56)	49 (42–57)	46 (39–55)	0.393 ^e^
Duration of disease, years ^b^	13 (7–18)	15 (11.25–20.5)	12 (4–18)	0.103 ^e^
Age of onset, years ^b^	30 (22–43)	29.5 (21.25–43)	32 (23–43)	0.583 ^e^
Disease extension (E1/E2/E3 ^c^)	14/14/51	6/8/26	8/6/25	0.748 ^d^
Fecal calprotectin, µg/g ^b^	90.6 (36.7–391)	99.3 (42.9–660.5)	88.3 (32.2–309)	0.246 ^e^
Immunomodulators use ^a^	20 (25.3)	10 (25.0)	10 (25.6)	1.000 ^d^
5-ASA dose at the baseline, mg/day ^b^	3600 (3000–4000)	3600 (3100–4000)	3000 (2400–3600)	<0.01 ^e^
Increased dose of 5-ASA, mg/day ^b^	–	0	1200 (1000–1200)	–
Prior use of systemic steroid ^a^	30 (38.0)	16 (40.0)	14 (35.9)	0.818 ^d^
UCEIS score (total) ^b^	2 (1–2)	1.5 (1–2)	2 (1–2)	0.452 ^e^
Vascular subscore ^b^	1 (0–1)	1 (1–2)	1.5 (1–2)	0.261 ^e^
Bleeding subscore ^b^	0 (0–1)	0 (0–1)	0 (0–1)	0.808 ^e^
Erosion/ulcer subscore ^b^	0 (0–0)	0 (0–0)	0 (0–0)	0.675 ^e^

Patient characteristics in the full analysis set (*n* = 79) at baseline. Data are shown as ^a^number (%), ^b^median (interquartile range) and ^c^number. Disease extension is classified according to the Montreal classification: E1, proctitis; E2, left-sided colitis; and E3: extensive colitis.

Characteristics between control and therapeutic intervention groups were compared and analyzed with ^d^Fisher exact test or ^e^Mann-Whitney *U* test.

Abbreviations: 5-ASA, 5-aminosalicylic acid; UCEIS, ulcerative colitis endoscopic index of severity.

Of the 79 patients in the FAS, 46 (58.2%) were male, and 20 (25.3%) were being treated with immunomodulators. Median fecal calprotectin was 90.6 µg/g (IQR, 36.7-391), and the median dose of 5-ASA was 3600 mg/day (IQR, 3000-4000). The daily dose of 5-ASA at baseline was higher in the control group (median [IQR], 3600 mg/day [3100-4000]) than the therapeutic intervention group (3000 mg/day [2400-3600]; *P* < .01). None of the other characteristics was significantly different between the 2 groups (Table 1). The median increased dose of 5-ASA (IQR) in the therapeutic intervention group was 1200 mg/day (1000-1200). Baseline characteristics of patients treated with the maximum dose of 5-ASA in its formulation at baseline and those administered a nonmaximum dose are presented in Supplementary Table 3. Although the distribution of disease extension was different between the maximum dose and nonmaximum dose groups, the difference was not statistically significant between the control and therapeutic intervention groups in the FAS (*P* = .748; Table 1).

### Efficacy of the Therapeutic Intervention: Primary Analysis

**Figure 2.**
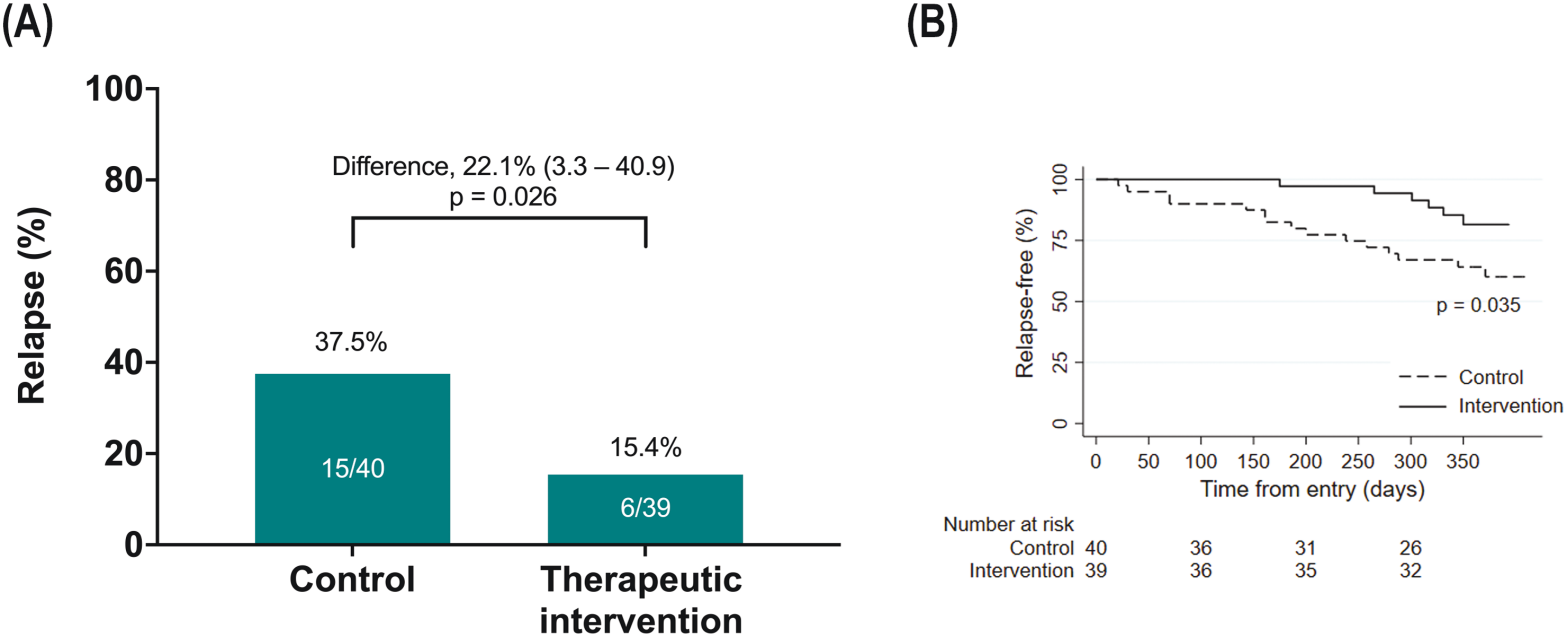
Comparison of relapse proportions between the therapeutic intervention and control groups. A, The relapse proportions within 1 year (primary end point) in the intention-to-treat analysis. The difference in relapse proportions between the 2 groups (95% confidence interval) is shown. The χ^2^ test was used for the analysis. B, Kaplan–Meier plots of cumulative relapse-free survival in the 2 groups. The log-rank test was used for the analysis.

The relapse proportion within 1 year was 15.4% (95% CI, 5.9-30.5) in the therapeutic intervention group and 37.5% (95% CI, 22.7-54.2) in the control group (Figure 2A). The relapse was less in the therapeutic intervention group (*P* = .026), and the absolute risk reduction (ARR) was 22.1% (95% CI, 3.3-40.9). Thus, the number needed to treat was 4.52. The cumulative relapse-free proportions are depicted in Kaplan–Meier plots (Figure 2B), and they were higher in the therapeutic intervention group than in the control group (*P* = .035). The cumulative relapse rate was 45.9 per 100 person-years in the control group and 17.4 per 100 person-years in the therapeutic intervention group.

### Sensitivity Analyses

We conducted 2 sensitivity analyses. First, the stratification factor (the use of immunomodulators) was adjusted with the Cochran–Mantel–Haenszel method, and the therapeutic intervention group had a lower odds of relapse than the control group (odds ratio 0.31; 95% CI, 0.11-0.90; *P* = .025). This finding was similar to that of the primary analysis. Second, another definition of relapse based on 2-item patient-reported outcomes regardless of the additional induction therapy was applied. In this analysis, the proportion of relapse within 1 year was reduced in the therapeutic intervention group (20.5%; 95% CI, 9.3-36.5) compared with that in the control group (42.5%; 95% CI, 27.0-59.1; *P* = .036). This finding was also similar to that of the primary analysis (Supplementary Figure 1).

### Medication Adherence

Data on median medication adherence were available in 77 patients (missing data in 2 patients), and it was 97.3% (IQR, 93.1-100) in the control group (*n* = 38) and 98.3% (IQR, 92-100) in the therapeutic intervention group (*n* = 39).

### Subgroup Analysis: Use of Immunomodulators

**Figure 3.**
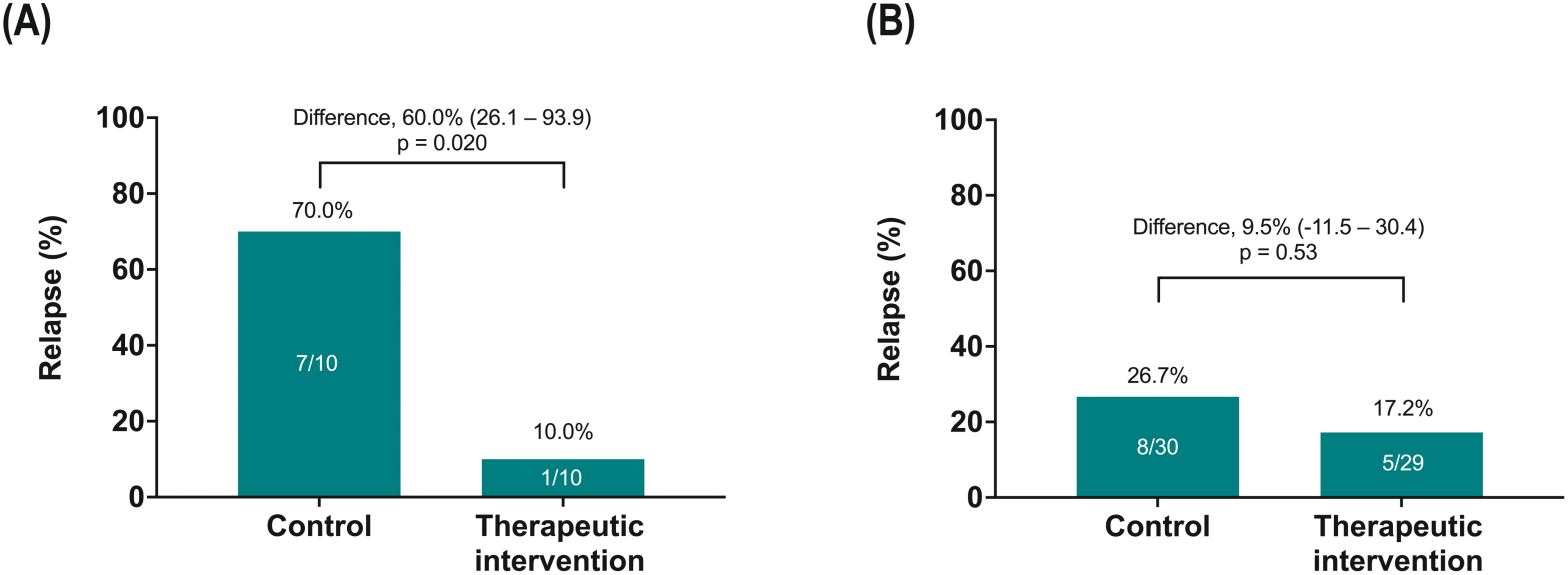
Subgroup analyses stratified for use of immunomodulators. The relapse proportion within 1 year in patients treated (A) with or (B) without concomitant immunomodulators. The difference in relapse proportions between the therapeutic intervention and the control groups (95% confidence intervals) are shown. The Fisher exact test was used for the analyses.

Subgroup analyses of patients with or without concomitant immunomodulators were conducted. In the subgroup with immunomodulators (*n* = 20), the characteristics were comparable between the 2 groups (Supplementary Table 4), and the relapse proportion in the therapeutic intervention group (10.0%; 95% CI, 0.25-44.5) was less than that in the control group (70.0%; 95% CI, 34.8-93.3; *P* = .020; Figure 3A). The dose (calculated as azathioprine) and the breakdown of immunomodulators are presented in Supplementary Table 5. Among patients not treated with immunomodulators (*n* = 59), the difference in the relapse proportion due to therapeutic intervention was not statistically significant (*P* = .53; Figure 3B).

### Power Analyses

Post hoc power analyses were conducted to evaluate the actual power and internal validity. In the analysis for entire patients (primary analysis), the calculated power was 0.61. Regarding the subgroup analysis, the calculated power was 0.67 in the patients with immunomodulators (*n *= 20) and 0.09 in those without immunomodulators (*n* = 59).

### Safety Assessment

Adverse events potentially associated with UC treatment developed in 3 patients in the therapeutic intervention group. Two patients complained of nausea, and one of these patients had been changed to a different 5-ASA formulation for dose escalation. Another patient complained of diarrhea and melena after switching to a different formulation of 5-ASA for dose escalation. Both patients improved by returning to the original dosage and former formulation. There were no severe adverse events.

Other adverse events, which were not likely to be associated with treatment or UC itself, were found in both groups. In the therapeutic intervention group, one patient caught influenza and another patient caught a common cold. In the control group, one patient caught a common cold, and another patient had an incidental computed tomography finding suspicious for bronchiolitis, which had disappeared on follow-up computed tomography 3 months later.

### Additional Treatment for Relapse

Thirteen patients started topical agents for relapse, including 4 in the intervention group and 9 in the control group. Two patients in the control group started a topical agent together with dose escalation of oral 5-ASA. Systemic steroid treatment was started in 3 patients, including 2 in the therapeutic intervention group and 1 in the control group (Table 2).

**Table 2.** Additional treatments for relapse.

Additional Treatment	Total *n* = 21	Control *n* = 15	Therapeutic Intervention *n* = 6
Topical treatment	13	9	4
Increasing dose of 5-ASA and topical treatment	2	2	0
Granulocyte and monocyte adsorptive apheresis	1	1	0
Systemic steroid	3	2	1
Tofacitinib	1	1	0
Other	1	0	1

Data are shown as absolute numbers of patients. Abbreviation: 5-ASA, 5-aminosalicylic acid.

### Predictive Factors for Relapse

**Figure 4.**
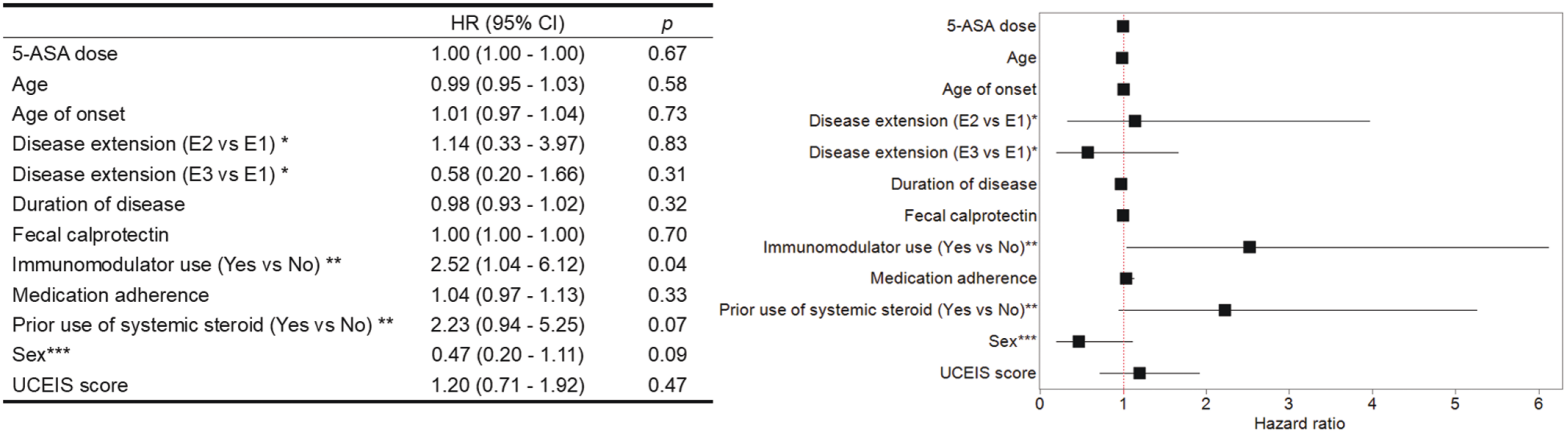
Exploratory analysis of predictors for relapse. Estimated hazard ratios and 95% confidence intervals for relapse (left). The results are presented in the Forest plot (right). Cox proportional hazard models were used for the estimation. The therapeutic intervention and each of the candidate predictors were adjusted in the analyses. Error bars shows the 95% confidence intervals. Disease extension was classified according to the Montreal classification: E1, proctitis; E2, left-sided colitis; and E3, extensive colitis. ^*^E1 as reference, ^**^no as reference, ^***^female as reference. Abbreviations: 5-ASA, 5-aminosalicylic acid; UCEIS, ulcerative colitis endoscopic index of severity.

Exploratory analyses investigating the predictors for relapse revealed that use of immunomodulators was predictive for relapse (adjusted hazard ratio 2.52; 95% CI, 1.04-6.12; *P* = .041), whereas all the other variables including medication adherence were not identified as predictors for relapse (Figure 4).

### Differences in Therapeutic Intervention and Relapse

To explore whether the change in 5-ASA formulation affected the risk of relapse, a subgroup analysis of patients with therapeutic intervention (*n* = 39) was conducted. Of 39 patients, the 5-ASA formulation was changed in 17 patients. The relapse proportions within 1 year were 11.8% (2 o f17) and 18.2% (4 of 22) in those with or without a change in 5-ASA formulation, respectively (*P* = .68). In a univariable logistic regression analysis, 5-ASA formulation change was not a predictive factor for relapse (OR, 0.60; 95% CI, 0.01-3.74; Supplementary Table 6). The association between an increment in the dose of 5-ASA (Δ5-ASA) and relapse was also investigated. Patients were categorized into 2 groups: Δ5-ASA ≤1000 mg/day (*n* = 15) and Δ5-ASA >1000 mg/day (*n* = 24). In a univariable logistic regression analysis, Δ5-ASA was not predictive for relapse (OR, 3.68; 95% CI, 0.39–35.14; Δ5-ASA ≤1000 mg/day as a reference; Supplementary Table 6).

## Discussion

This study showed that dose escalation of 5-ASA in UC patients in clinical remission with an MES of 1 reduced the risk of relapse within 1 year. The ARR was 22.1%, and the number needed to treat was 4.52. In our previous retrospective study, the ARR of therapeutic intervention for UC patients with an MES of 1 was approximately 20%, which is roughly consistent with the ARR in this study.^11^

Whether all patients with an MES of 1 should receive therapeutic intervention with a dose escalation of 5-ASA is a relevant issue in terms of cost-effectiveness. In the current study, concomitant use of immunomodulators was identified as a predictor for relapse. Furthermore, the efficacy of the therapeutic intervention was evident in patients using concomitant immunomodulators but not in those not treated with immunomodulators. Patients concomitantly treated with immunomodulators may be more likely to have refractory UC, such as steroid-dependent UC. Systemic steroids had been used in 65.0% (13 of 20) of patients concomitantly treated with immunomodulators but only 28.8% (17 of 59) of patients not treated with immunomodulators (Supplementary Table 1). Thus, more than 70% of patients not using immunomodulators had reached remission without systemic steroids and had maintained remission with an oral 5-ASA agent. It is possible that the dose escalation of 5-ASA was more efficacious in those with more refractory diseases, such as patients requiring concomitant immunomodulators.

In the subgroup analysis with concomitant immunomodulators, 70% of the patients relapsed in the control group, which was higher than that in the therapeutic intervention group (10%). The high proportion of relapses in the control group might have led to the findings of this subgroup analysis. Patients who require concomitant immunomodulators to achieve an MES of 1 might be at higher risk for relapse due to refractory disease than those without immunomodulators, and it was possible that the therapeutic effect of the dose escalation of 5-ASA was greater for patients with concomitant immunomodulators than those without. However, the number of patients was small (*n* = 10, in each group). Therefore, these patients might not be representative of the general population. Nonetheless, the patient characteristics were comparable between the control and therapeutic intervention groups in the subgroup with immunomodulators (Supplementary Table 4) owing to randomization using the stratification factor of thiopurine use. Notably, the parameters representative of disease activity, such as fecal calprotectin and UCEIS scores, were also comparable between the 2 groups and within the range of quiescent disease. Unmeasurable potential risk factors for relapse might have caused bias of the results to some extent because of the small sample size, even after randomization by stratification of immunomodulator use. These findings warrant further research with a large number of patients to investigate the generalizability of the therapeutic efficacy under treatment with immunomodulators.

In the present study, the hazard ratio for relapse for prior use of systemic steroids was 2.23 (95% CI, 0.94-5.25; *P* = .068) after adjustment for the therapeutic intervention. Although our study did not reveal the risk of prior use of steroids for relapse, a recent study reported that prior use of systemic steroid was a risk factor for clinical relapse in patients with an MES of 1.^17^ The possible reason for this discrepancy could be related to the small sample size of the current study. Further research is needed to clarify which UC patients in clinical remission with an MES of 1 would benefit most from dose escalation of 5-ASA.

Regarding the difference in therapeutic intervention (ie, change in 5-ASA formulation, increment in the daily dose of 5-ASA), subgroup analyses of patients with therapeutic intervention were conducted. As a result, neither changing 5-ASA formulation nor Δ5-ASA >1000 mg/day were predictive for relapse (Supplementary Table 6). However, only a univariable analysis could be conducted because of the small number of relapses; thus, further research, including multivariable analysis with a sufficiently large sample size, is needed to explore the association between the impact of changing the brand of 5-ASA and the reduction of future relapse.

In recent years, the concept of clinical inertia has been attracting attention. For example, the phenomenon of clinical inertia in diabetology is defined as the failure to start a therapy or to optimize therapy when appropriate. Clinical inertia prevents good glycemic control in patients with diabetes.^18^ Clinical inertia is also a focus of attention in inflammatory bowel disease practice, and it is important not to avoid intervening in the case of patients with an MES of 1 who are at high risk for relapse.

Three adverse events were potentially associated with the therapeutic intervention in this study. One patient complained of acute aggravation of melena and diarrhea after a change of 5-ASA formulation and dose escalation. The symptoms improved after the patient returned to the former dose and formulation, which suggests that this adverse event was 5-ASA intolerance. This intolerance can be influenced by the mesalamine release characteristics of each formulation, such as specific coatings or additives.^19^ Even when patients are tolerant to one formulation of 5-ASA, they may be intolerant to another 5-ASA formulation.^20^ Therefore, care should be taken when changing patients’ 5-ASA formulations.

There were several limitations in our study. First, it was not a double-blind study. When patients relapsed, it was at the discretion of the physician whether to initiate therapeutic intervention. Thus, the outcome might be biased. To reduce this bias, we conducted a sensitivity analysis using the definition of relapse with the 2-item patient-reported outcomes score, which consists of only objective measures. We found that the relapse was less in the therapeutic intervention group (20.5%; 95% CI, 9.3-36.5) than the control group (42.5%; 95% CI, 27.0-59.1; *P* = .036). This result suggests that the decision to intervene in treatment did not affect the result of the primary end point. In addition, the placebo effect related to the unblinded design might have led to the overestimation of the therapeutic efficacy in the therapeutic intervention group. To overcome this issue related to the unblinded design, a double-blind randomized controlled trial is required. Second, the final sample size (*n* = 79) was smaller than that originally designed (*n* = 220). The post hoc power analysis estimated the actual power lower than 0.80, meaning that type II error could be increased. Nevertheless, the present study revealed that therapeutic intervention reduced the relapse significantly among all the patients and in the subgroup with immunomodulators. The result in those without immunomodulators could not be interpreted exactly due to extremely low power. Regarding the sample size, there might have been unadjusted potential confounders. Indeed, the 5-ASA dose at baseline was statistically different between the therapeutic intervention group and the control group. Some unmeasured or unmeasurable potential risk factors for relapse might be confounding, even after randomization. Third, endoscopic evaluations were not conducted at the end of the study. Therefore, it could not be determined whether MES improved from 1 to 0 with therapeutic intervention. Fourth, the effect of changing the 5-ASA formulation was not evaluated. However, a previous study has suggested that differences in drug delivery systems do not affect therapeutic efficacy.^21^ Finally, independent randomization was carried out in all the institutions but not in each hospital. Therefore, the difference in characteristics such as refractoriness or clinical practice might be potentially confounding. To examine the difference in clinical practice for relapse in patients in different hospitals, we conducted a sensitivity analysis based on 2-item patient-reported outcomes regardless of adding treatment, and similar results were obtained.

In conclusion, dose escalation of oral 5-ASA reduced the risk of relapse within 1 year in patients with UC in clinical remission with an MES of 1.

## Supplementary Data

Supplementary data is available at *Inflammatory Bowel Diseases* online.

izae088_suppl_Supplementary_Material

## Abbreviations:

UC: ulcerative colitis
MES: Mayo endoscopic subscore
5-ASA: 5-aminosalicylic acid
FAS: full analysis set
IQR: interquartile range
ARR: absolute risk reduction
