# Supplementary material for: Efficacy of Dose Escalation of Oral 5-Aminosalicylic Acid for Ulcerative Colitis With a Mayo Endoscopic Subscore of 1: An Open Label Randomized Controlled Trial
Source: Inflamm Bowel Dis. 2024 Apr 24;31(3):716–24. doi: 10.1093/ibd/izae088 (PMC11879217; doi:10.1093/ibd/izae088)
Supplement: izae088_suppl_Supplementary_Material [file izae088_suppl_supplementary_material.docx]

**Supplementary Table 1.** Concomitant use of immunomodulators and prior use of systemic steroid.

| Prior use of systemic steroid | Total  n = 79 | Concomitant use of IM  n = 20 | Without use of IM  n = 59 |
| --- | --- | --- | --- |
| Yes | 30 (38.0) | 13 (65.0) | 17 (28.8) |
| No | 49 (62.0) | 7 (35.0) | 42 (71.2) |

Data are shown as number (%). IM, immunomodulators.

**Supplementary Table 2.** Detailed information on 5-ASA agent used at the baseline.

| 5-ASA | Control  n = 40 | Therapeutic intervention  n = 39 |
| --- | --- | --- |
| Time dependent mesalazine |  |  |
| 1500mg | 0 | 1 |
| 2000mg | 0 | 2 |
| 2250mg | 1 | 2 |
| 2400mg | 0 | 1 |
| 3000mg | 2 | 3 |
| 3600mg | 0 | 1 |
| 4000mg | 12 | 8 |
| pH dependent mesalazine |  |  |
| 2400mg | 2 | 5 |
| 3200mg | 2 | 0 |
| 3600mg | 13 | 8 |
| Multi Matrix system mesalazine | 0 | 0 |
| Salazosulfapyridine |  |  |
| 1000mg | 0 | 1 |
| 1500mg | 0 | 1 |
| 2000mg | 0 | 1 |
| 3000mg | 5 | 5 |
| 4000mg | 3 | 0 |
|  |  |  |

Absolute numbers of patients are shown with respect to each dose or formulation of 5-ASA.

**Supplementary Table 3. Patient characteristics stratified by baseline 5-ASA dose.**

| Characteristic | non-Maximum  n = 36 | Maximum  n = 43 | p value |
| --- | --- | --- | --- |
| Male ^a^ | 21 (58.3) | 25 (58.1) | 1.000 ^d^ |
| Age, years ^b^ | 48 (42–54) | 49 (38–56) | 0.854 ^e^ |
| Duration of disease, years ^b^ | 15 (11–18) | 13 (4–19) | 0.303 ^e^ |
| Age of onset, years ^b^ | 30 (24.5–42) | 32 (20–45) | 0.858 ^e^ |
| Disease extension (E1/E2/E3 ^c^) | 23/3/10 | 28/11/4 | 0.040 ^d^ |
| Fecal calprotectin, µg/g ^b^ | 154.0  (36.7–444.0) | 75.7  (37.2–361.0) | 0.551 ^e^ |
| Immunomodulators use ^a^ | 8 (22.2) | 12 (27.9) | 0.612 ^d^ |
| 5-ASA dose at the baseline,  mg/day ^b^ | 3000  (2325–3000) | 3600  (3600–4000) | <0.01 ^e^ |
| Prior use of systemic steroid ^a^ | 12 (33.3) | 18 (41.9) | 0.491 ^d^ |
| UCEIS score (total) ^b^ | 2 (1–2.5) | 2 (1–2) | 0.602 ^e^ |
| Vascular subscore ^b^ | 1 (1–2) | 1 (1–2) | 0.426 ^e^ |
| Bleeding subscore ^b^ | 0 (0–1) | 0 (0–1) | 0.405 ^e^ |
| Erosion/ulcer subscore ^b^ | 0 (0–0) | 0 (0–0) | 0.557 ^e^ |

Patient characteristics stratified by baseline 5-ASA dose (Maximum dose or not) are presented. Data are shown as ^a^ number (%), ^b^ median (interquartile range) and ^c^ number. Disease extension is classified according to the Montreal classification: E1, proctitis; E2, left-sided colitis; and E3: extensive colitis. Each characteristic was compared between the two groups with compared and analyzed with ^d^ Fisher’s exact test or ^e^ Mann-Whitney *U* test.

5-ASA, 5-aminosalicylic acid; UCEIS, ulcerative colitis endoscopic index of severity.

**Supplementary Table 4.** **Patient characteristics in the subgroup under treatment with concomitant immunomodulators.**

| Characteristic | Total  n = 20 | Control  n = 10 | Therapeutic intervention  n = 10 | p value |
| --- | --- | --- | --- | --- |
| Male ^a^ | 12 (60.0) | 4 (40.0) | 8 (80.0) | 0.170 ^d^ |
| Age, years ^b^ | 44.5 (36–50.5) | 47 (37–50) | 42.5 (32–51) | 0.520 ^e^ |
| Duration of disease, years ^b^ | 18 (14–26) | 18.5 (15–26) | 18 (12–26) | 1.000 ^e^ |
| Age of onset, years ^b^ | 22 (19–27) | 24.5 (19–34) | 19.5 (19–27) | 0.492 ^e^ |
| Disease extension (E1/E2/E3 ^c^) | 1/2/17 | 1/1/8 | 0/1/9 | 1.000 ^d^ |
| Fecal calprotectin, µg/g ^b^ | 88.3 (29.2–191) | 99.3 (44.8–184) | 71.5 (29.2–191) | 0.513 ^e^ |
| 5-ASA dose at the baseline,  mg/day ^b^ | 3600  (3000–4000) | 3600  (3200–4000) | 3300  (3000–3600) | 0.193 ^e^ |
| Increased dose of 5-ASA,  mg/day ^b^ | – | 0 | 1100  (800–1200) | – |
| Prior use of systemic steroid ^a^ | 13 (65.0) | 7 (70.0) | 6 (60.0) | 1.000 ^d^ |
| UCEIS score (total) ^b^ | 1 (1–2) | 1 (1–2) | 2 (1–3) | 0.287 ^e^ |
| Vascular subscore ^b^ | 1 (1–2) | 1 (1–1) | 2 (1–2) | 0.118 ^e^ |
| Bleeding subscore ^b^ | 0 (0–1) | 0 (0–0) | 0 (0–1) | 0.098 ^e^ |
| Erosion/ulcer subscore ^b^ | 0 (0–0) | 0 (0–0) | 0 (0–0) | 0.342 ^e^ |

Patient characteristics in the subgroup under treatment with immunomodulators (n = 20) at the baseline. Data are shown as ^a^ number (%), ^b^ median (interquartile range) and ^c^ number. Disease extension is classified according to the Montreal classification: E1, proctitis; E2, left-sided colitis; and E3: extensive colitis.

Characteristics between control and therapeutic intervention groups were compared and analyzed with ^d^ Fisher’s exact test or ^e^ Mann-Whitney *U* test.

5-ASA, 5-aminosalicylic acid; UCEIS, ulcerative colitis endoscopic index of severity.

**Supplementary Table 5. Detailed information about thiopurine use.**

| Thiopurines | Control  n = 10 | Therapeutic intervention  n = 10 | p value |
| --- | --- | --- | --- |
| Azathioprine ^a^ | 6 (60.0) | 4 (40.0) | – |
| 6-mercaptopurine ^a^ | 4 (40.0) | 6 (60.0) | – |
| Thiopurine dose, mg/kg/day ^b^ | 1.18 (0.91–1.48) | 1.22 (0.58–1.35) | 0.820 ^c^ |

The absolute numbers and proportions of azathioprine or 6-mercaptopurine used in each of the groups are presented. In case of 6-mercaptopurine use, the dosage was divided by a coefficient of 2.08 and converted to the equivalent pharmaceutical dose of azathioprine. The daily dose of thiopurine was standardized by body weight.

Data are shown as ^a^ number (%) or ^b^ median (interquartile range).

^c^ Mann-Whitney *U* test.

**Supplementary Table 6.** Difference in the therapeutic intervention and relapse.

|  | **Crude analysis** | |
| --- | --- | --- |
|  | **OR (95% CI)** | ***p*** |
| 5-ASA formulation change ^a^ | 0.60 (0.96–3.74) | 0.58 |
| Δ5-ASA dose ^b^ |  |  |
| >1000 mg/day | 3.68 (0.39–35.14) | 0.26 |
| ≤1000 mg/day | reference |  |

Univariable logistic regression analysis for predicting relapse within one year.

^a^Patients not changed 5-ASA formulation as reference.

^b^Increased dose of 5-ASA as the therapeutic intervention.

5-ASA, 5-aminosalicylic acid; CI, confidence interval; OR, odds ratio.


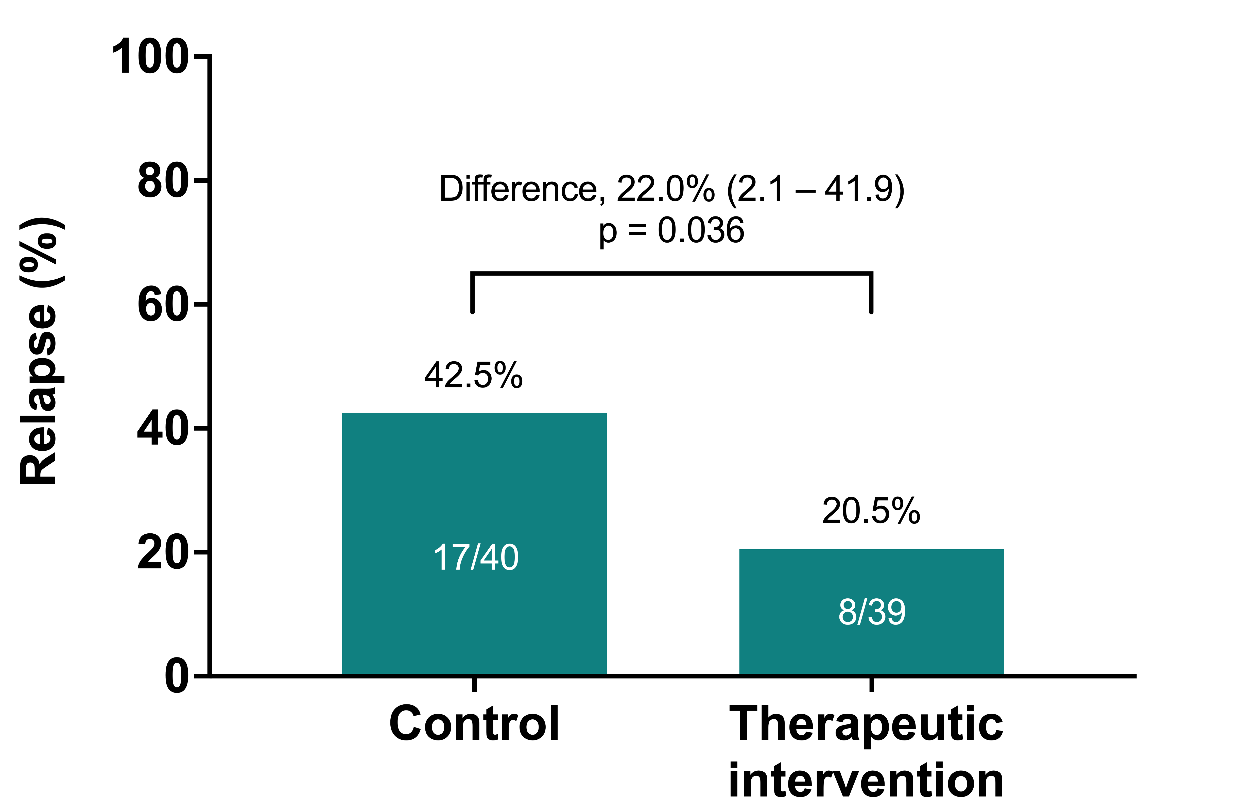
**Supplementary Figure 1**. **Sensitivity analysis for relapse in the therapeutic intervention and control groups.**

Relapse was defined based on two-item patient-reported outcomes (PRO2) in the sensitivity analysis. A rectal bleeding subscore ≥1 or stool frequency subscore ≥2 in PRO2, regardless of additional induction therapy, was treated as relapse. The difference in relapse rates between the two groups (95% confidence interval) is shown. The chi-square test was used for the analysis.

**Supplementary appendix**

Exclusion criteria in this study were as follows:

1) Complications of enteritis other than ulcerative colitis (for example, infectious enteritis)

2) Symptomatic intestinal stricture

3) Planning to undergo colorectal resection

4) Serious infectious diseases

5) Concurrent diagnosis with any malignant tumors

6) Treated with maintenance therapy other than oral 5-aminosalicylic acid and thiopurine

7) Deemed inappropriate for inclusion in the study by the investigators
